# Supplementary material for: 3D projection electrophoresis for single-cell immunoblotting
Source: Nat Commun. 2020 Dec 4;11:6237. doi: 10.1038/s41467-020-19738-1 (PMC7718224; doi:10.1038/s41467-020-19738-1)
Supplement: Supplementary file 1 — Supplementary Information [file 41467_2020_19738_MOESM1_ESM.pdf]

Supplementary information for:

3D projection electrophoresis for single-cell immunoblotting

Samantha M. Grist, Andoni P. Mourdoukoutas, and Amy E. Herr

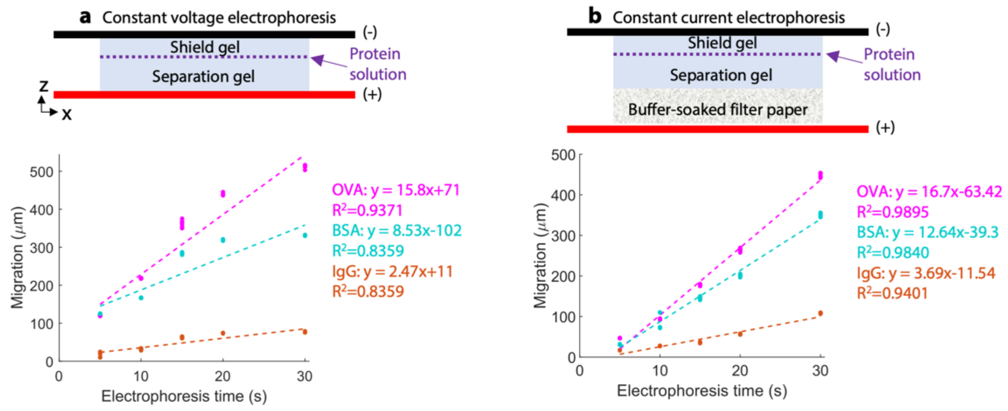

Supplementary Figure 1. Optimization of *z*-directional electrophoresis system to facilitate constant-velocity migration. **(a)** Shows a cross-sectional view of the setup before optimization. The separation gel is in direct contact with the anode, and the electric field was supplied as constant voltage. Linear fits to the migration data are poor, with migration slowing at increasing electrophoresis times. **(b)** Depicts the system after optimization. A buffer-soaked filter paper is placed between the separation gel and the anode to mitigate pH changes due to electrolysis at the electrode surface, and the electric field was supplied as constant current to mitigate changes in electrical resistance in series with the separation gel (due to bubble formation at the electrodes). Linear fits to the migration data are improved.

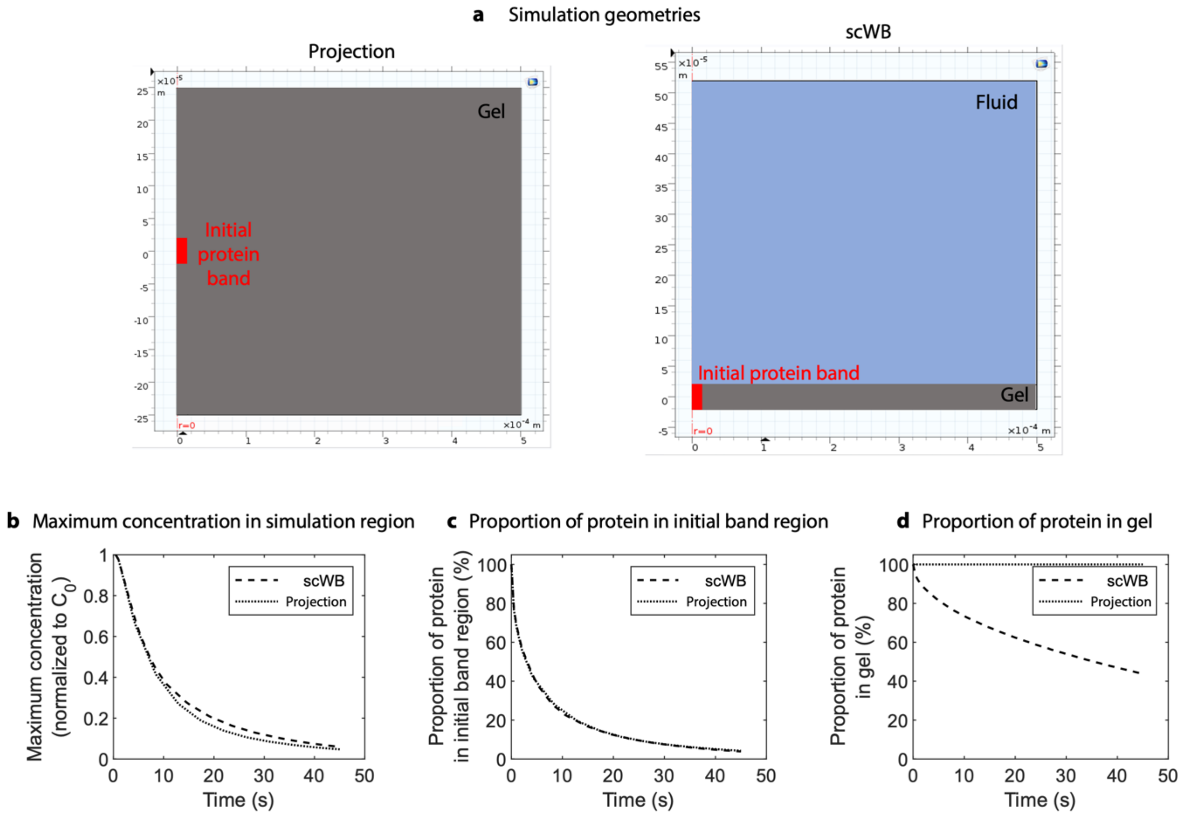

Supplementary Figure 2. Comparison of simulated in-gel protein dilution during electrophoresis for standard single-cell western blotting and *z*-direction electrophoresis. **(a)** 2D axisymmetric simulation geometries. **(b)** Comparison of maximum concentration in the simulation region, normalized to the initial concentration in the protein band (C_0). **(c)** Proportion of the protein in the initial simulation band region. **(d)** Proportion of protein retained in the gel.

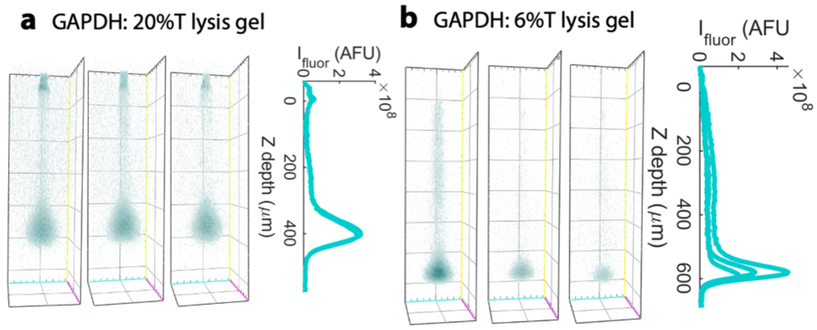

Supplementary Figure 3. Representative 3D renderings (left) and summed fluorescence *z*-intensity profiles (right) of GAPDH separations from BT474 breast tumour cells after lysis using **(a)** a 20%T lysis gel, and **(b)** a 6%T lysis gel, both using 2× RIPA + 8M urea lysis buffer and after 10s electrophoresis. By moving to 6%T lysis gels, we observed higher apparent GAPDH mobility (1.08±0.03×10^{-4} cm^2/V·s using 6%T lysis gel, compared with 0.83±0.08×10^{-4} cm^2/V·s using 20%T lysis gel, n = 12-14 separation lanes) and potential reduction in dispersion of the protein band towards the microwell.

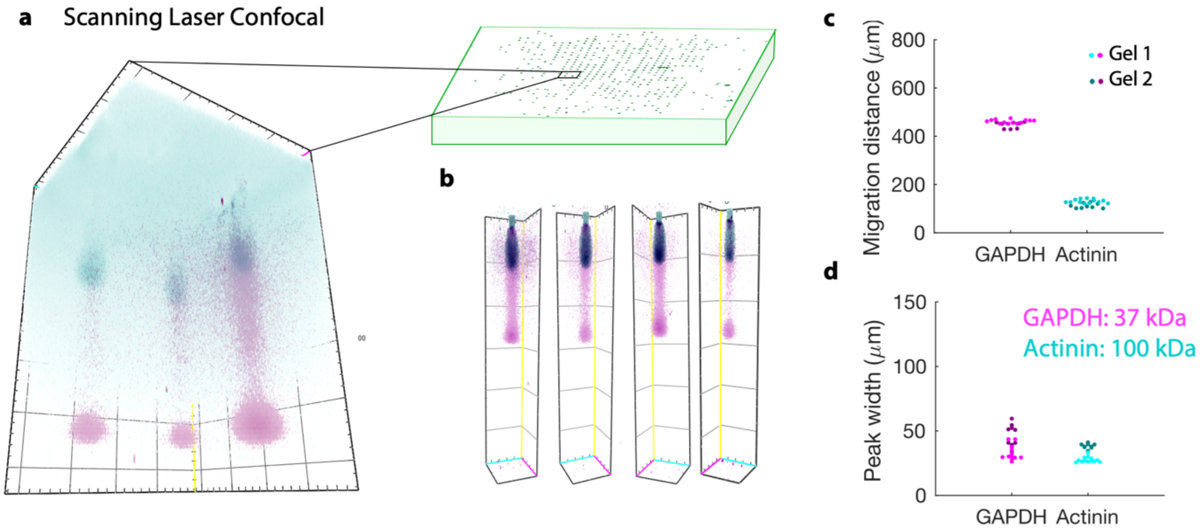

Supplementary Figure 4. Comparison imaging by scanning laser confocal microscopy of the same projection electrophoresis separation gels analyzed in Figure 5. **(a)** a scanning laser confocal field of view compared to the size of the separation gel. **(b)** maximum intensity projection 3D renderings of representative individual separation lanes read out by scanning laser confocal microscopy. **(c)** quantification of migration distance for GAPDH (37 kDa) and actinin (100 kDa). **(d)** quantification of *z*-directional peak width for the separated bands of the same protein targets. For (c-d), individual dots are plotted for two independent separation gels. Dots corresponding to n = 13 (gel 1) and n = 9 (gel 2) separation lanes are plotted.

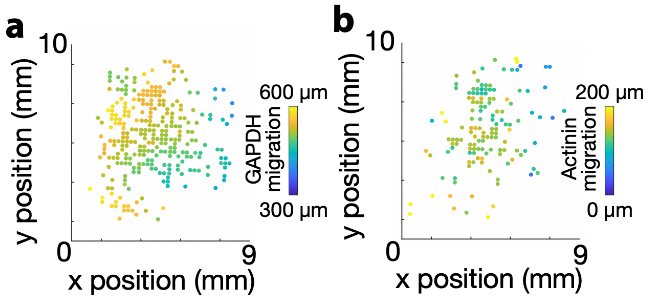

Supplementary Figure 5. Spatial map of the variation in **(a)** GAPDH and **(b)** actinin electromigration distances across the *xy* gel area, for the duplicate separation gel (first gel is presented in Fig. 5n-o)

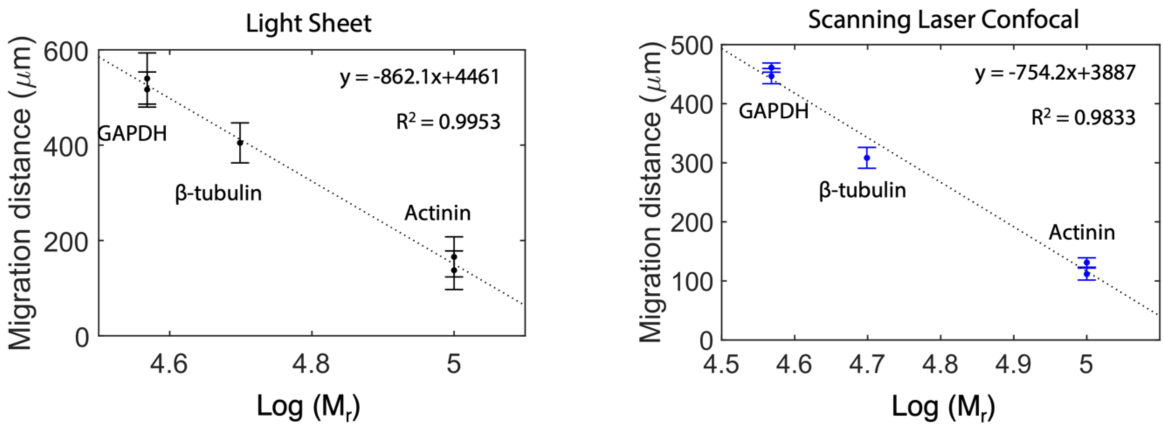

Supplementary Figure 6. Quantification distance of migration distance vs. log(molecular mass) for three endogenous protein targets measured from single BT474 breast tumour cells using light sheet (left) and scanning laser confocal (right) microscopy readouts for the Projection Electrophoresis assay. Both readout methods show the expected log-linear relationship affirming size separation. Each point plots the mean and standard deviation of quantifiable separation lanes from a single separation gel (duplicate gels for GAPDH and actinin; a single gel for β-tubulin). For the light sheet analysis, 100-300 separation lines were quantified to yield each plotted point; for the scanning laser confocal analysis, 9-13 separation lanes were quantified to yield each plotted point. For this experiment, β-tubulin was electrophoretically probed with Ms anti-β-tubulin primary (GeneTex GTX11312, 1:15 dilution) and Dk anti-Ms AlexaFluor 647 secondary (Invitrogen A31571, lot 2045337, 1:10 dilution) antibodies.

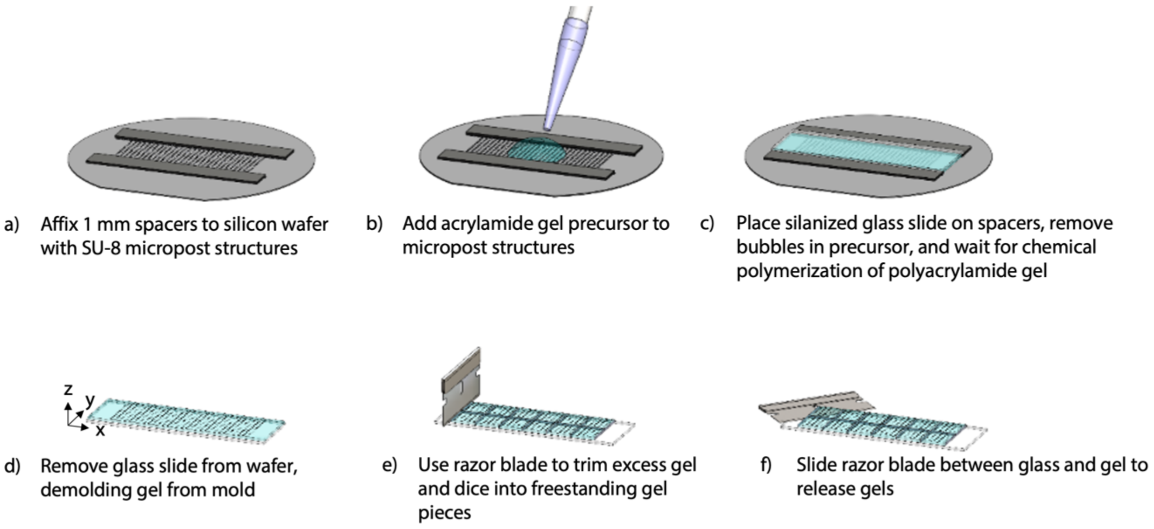

Supplementary Figure 7. Substrate-free released gel fabrication process.

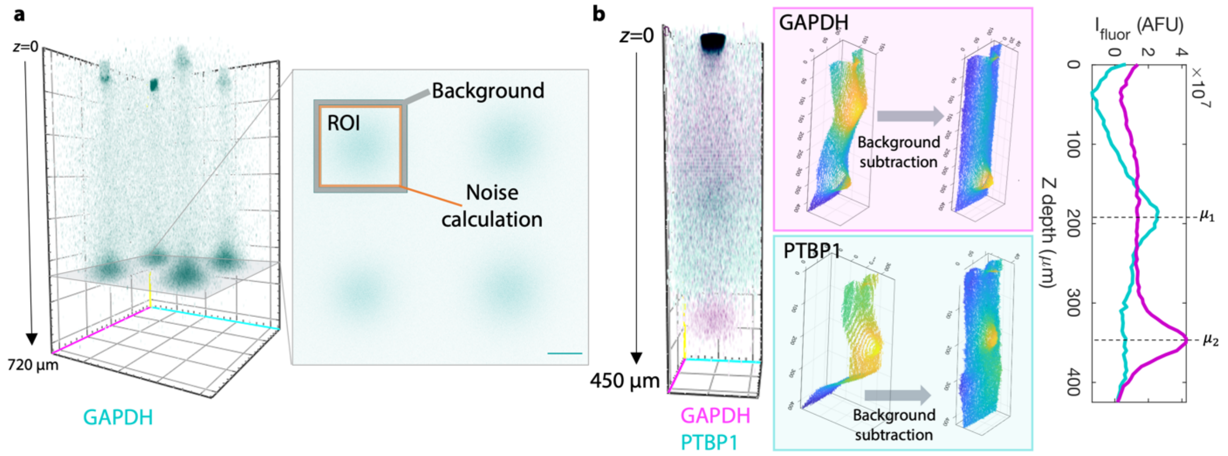

Supplementary Figure 8. Data processing for three-dimensional projection electrophoresis datasets from immunoprobed separations from single BT474 cells. **(a)** 3D data is composed of stacks of *xy* slice images. Each slice image is processed to isolate the region of interest ('ROI') for each separation lane, as well as small adjacent surrounding regions for background subtraction ('Background') and to calculate the noise of the background-subtracted signal ('Noise calculation'). Scale bar represents 50 µm. **(b)** The ROI corresponding to each separation lane is also a 3D dataset (rendering, left), which can be collapsed into a 2D (*xz*) image by summing in *y* (middle), or collapsed into a summed 1D intensity profile by summing all pixels in *x* and *y* within the ROI region (right). Subtraction of the average background region intensity at each *z*-depth from each pixel in the ROI (middle column) isolates the signal from the single-cell separation, clearly showing separated, immunoprobed protein peaks corresponding to GAPDH (top) and PTBP1 (bottom). Gaussian fitting to the background subtracted 1D intensity profiles yields migration distance, peak width, and signal-to-noise ratio information for each separation lane.

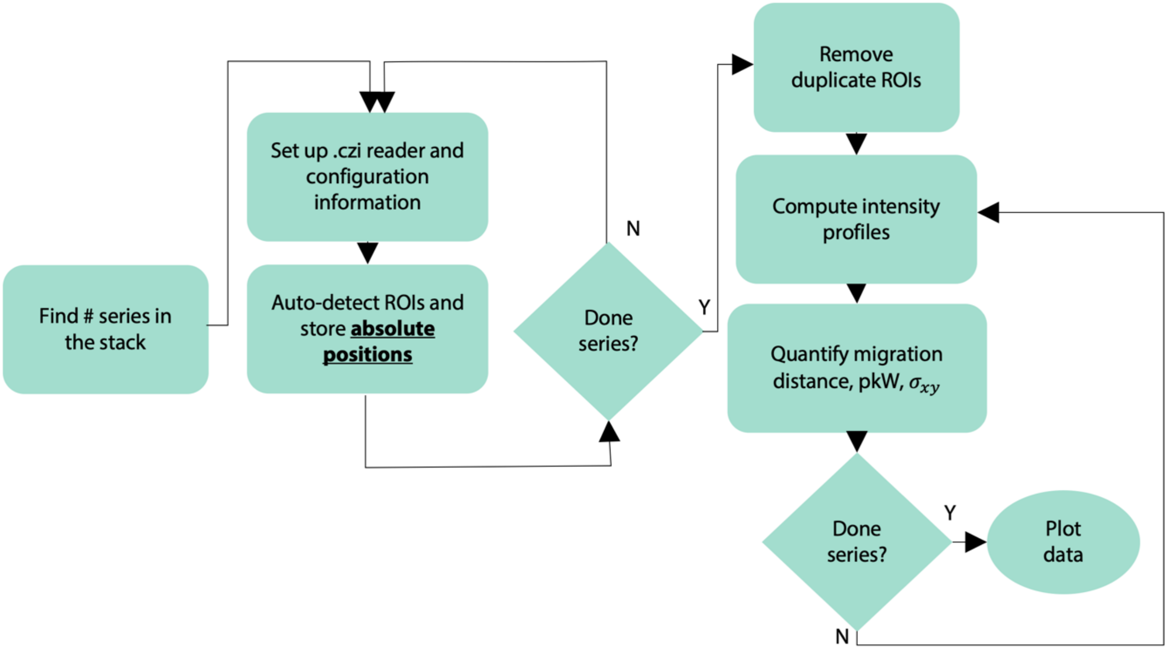

Supplementary Figure 9. Schematic diagram of image analysis software for tiled light sheet images.

Supplementary Table 1. Fabrication conditions for the various types of polyacrylamide gels used in this work.

Gel type (thickness)	Gel density	Stock acrylamide	Rhinohide	BPMA	Buffer	Initiator(s)	Polymerization time	Fabrication setup
Purified protein: separation (1 mm)	7%T or 10%T	30% (37.5:1) stock; final concentration 7% or 10% (w/v) Sigma-Aldrich: A3699	10% (v/v) final from stock	3 mM from 100 mM stock in DMSO	10% (v/v) final concentration 10× tris-glycine; stored in modified RIPA	0.08% (w/v) APS (Sigma-Aldrich: A3678), 0.08% (v/v) TEMED (Sigma-Aldrich: T9281)	60 mins	Methacrylate functionalized glass slide and silanized silicon wafer mould
Purified protein: shield (500 µm)	20%T	30% (37.5:1) stock; final concentration 20% (w/v)	10% (v/v) final from stock	none	10% (v/v) final concentration 10× tris-glycine; stored in run buffer (1× tris-glycine containing 0.5% Triton X-100)	0.08% (w/v) APS, 0.08% (v/v) TEMED	60 mins	Gel Slick® (Lonza: 50640) treated glass plate and glass slide
Well gels for single cell separations and in-well lysis tests (1 mm)	7%T	30% (37.5:1) stock; final concentration 7% (w/v)	4.66% (v/v) final concentration from stock	3 mM from 100 mM stock in DMSO	10% (v/v) final concentration 10× tris-glycine; stored in PBS (lysis monitoring) or 1× tris-glycine (single cell separations)	0.08% (w/v) APS, 0.08% (v/v) TEMED	60 mins	Methacrylate functionalized glass slide and silanized silicon wafer mould
Lysis shield gels for in-well lysis tests (1 mm)	20%T	30% (37.5:1) stock; final concentration 20% (w/v)	10% (v/v) final from stock	none	None; stored in modified RIPA	0.08% (w/v) APS, 0.08% (v/v) TEMED	60 mins	Gel Slick® treated glass plate and glass slide
Lysis shield gels for single-cell separations (1 mm)	6%T or 20%T	30% (37.5:1) stock; final concentration 6% or 20% (w/v)	10% (20%T) or 4.66% (6%T) (v/v) final from stock	none	10% (v/v) final concentration 10× tris-glycine; stored in 2× modified RIPA and transferred to 2× RIPA containing 8M urea for >10 minutes prior to separation.	0.08% (w/v) APS, 0.08% (v/v) TEMED	60 mins	Gel Slick® treated glass plate and glass slide

Supplementary Table 2. Projection electrophoresis buffers.

**1× tris-glycine and 0.5% Triton X-100**	**1× tris-glycine**	**1× RIPA and 1× tris-glycine**	**2× RIPA and 2× tris-glycine**	**2× RIPA and 8M Urea**
10× tris-glycine: 10% (v/v) Bio-Rad 1610734	10× tris-glycine: 10% (v/v) Bio-Rad 1610734	10× tris-glycine: 10% (v/v) Bio-Rad 1610734	10× tris-glycine: 20% (v/v) Bio-Rad 1610734	Urea: 8M final (Sigma-Aldrich U5378)
Triton X-100: 0.5% (v/v) Sigma-Aldrich: X100	MilliQ Water: 90% (v/v)	SDS: 0.5% (w/v) Sigma-Aldrich #L3771	SDS: 1% (w/v) Sigma-Aldrich #L3771	10× tris-glycine: 20% (v/v) Bio-Rad 1610734
MilliQ Water: 89.5% (v/v)	--	Sodium Deoxycholate: 0.25% (w/v) Sigma-Aldrich #D6750	Sodium Deoxycholate: 0.5% (w/v) Sigma-Aldrich #D6750	SDS: 1% (w/v) Sigma-Aldrich #L3771
--	--	Triton X-100: 0.1% (v/v) Sigma-Aldrich: X100	Triton X-100: 0.2% (v/v) Sigma-Aldrich: X100	Sodium Deoxycholate: 0.5% (w/v) Sigma-Aldrich #D6750
--	--	MilliQ Water: 89.9% (v/v)	MilliQ Water: 79.8% (v/v)	Triton X-100: 0.2% (v/v) Sigma-Aldrich: X100
--	--	--	--	MilliQ Water: 79.8% (v/v)

Supplementary Note 1. Consideration of refractive index mismatch on measured *z*-location in confocal microscopy.

One consideration when comparing light sheet and confocal microscopy is refractive index mismatch between the gel and the objective immersion medium. In confocal microscopy, mismatch in sample vs. immersion medium refractive index results in distortion of the imaging point spread function, as well as distortion of the apparent scanned distance in *z*:

$$\Delta f = \frac{n_2}{n_1}\frac{\left[1-\left(\frac{n_1}{n_2}\sin\theta_1\right)^2\right]^{1/2}}{\cos\theta_1}\Delta z,$$

Where Δf is the distance in *z* scanned by the focal spot, Δz is the distance in *z* scanned by the objective, n_1 is the objective immersion medium, n_2 is the sample medium, and θ_1 is the angle at which the marginal rays from the objective approach the top of the sample[1]. Although this equation derived from geometric optics is helpful in qualitatively understanding the effects of refractive index mismatch, experimental data often do not follow this relationship (likely due to the high sensitivity to θ_1)[1]. In contrast, with light sheet microscopy we may not expect this distortion of apparent *z*-directional distances because the position of the sectioned focal plane is controlled by the physical position of the light sheet within the sample, rather than by the optics of the detection objective. From this distortion function, we would expect to measure migration distances from confocal images shorter than those measured with light sheet microscopy (as n_2, or the gel refractive index, is slightly higher than the water immersion medium n_1). Comparing Supplementary Fig. 4 and Figure 5**l**, we indeed observe that confocal migration distances (Supplementary Fig. 4) are shorter than those measured from light sheet (Figure 5l).

Supplementary References

1. Diaspro, A., Federici, F. & Robello, M. Influence of refractive-index mismatch in high-resolution three-dimensional confocal microscopy. *Appl. Opt.* **41**, 685–690 (2002).
