# Supplementary material for: 3D projection electrophoresis for single-cell immunoblotting
Source: Nat Commun. 2020 Dec 4;11:6237. doi: 10.1038/s41467-020-19738-1 (PMC7718224; doi:10.1038/s41467-020-19738-1)
Supplement: Supplementary file 2 — Reporting Summary [file 41467_2020_19738_MOESM2_ESM.pdf]

nature research

Corresponding author(s): Prof. Amy E. Herr

Last updated by author(s): Sep 25, 2020

Reporting Summary

Nature Research wishes to improve the reproducibility of the work that we publish. This form provides structure for consistency and transparency in reporting. For further information on Nature Research policies, see Authors & Referees and the Editorial Policy Checklist.

Statistics

For all statistical analyses, confirm that the following items are present in the figure legend, table legend, main text, or Methods section.

n/a	Confirmed	
☐	☒	The exact sample size (*n*) for each experimental group/condition, given as a discrete number and unit of measurement
☐	☒	A statement on whether measurements were taken from distinct samples or whether the same sample was measured repeatedly
☒	☐	The statistical test(s) used AND whether they are one- or two-sided<br>*Only common tests should be described solely by name; describe more complex techniques in the Methods section.*
☒	☐	A description of all covariates tested
☒	☐	A description of any assumptions or corrections, such as tests of normality and adjustment for multiple comparisons
☐	☒	A full description of the statistical parameters including central tendency (e.g. means) or other basic estimates (e.g. regression coefficient) AND variation (e.g. standard deviation) or associated estimates of uncertainty (e.g. confidence intervals)
☒	☐	For null hypothesis testing, the test statistic (e.g. *F*, *t*, *r*) with confidence intervals, effect sizes, degrees of freedom and *P* value noted<br>*Give P values as exact values whenever suitable.*
☒	☐	For Bayesian analysis, information on the choice of priors and Markov chain Monte Carlo settings
☒	☐	For hierarchical and complex designs, identification of the appropriate level for tests and full reporting of outcomes
☒	☐	Estimates of effect sizes (e.g. Cohen's *d*, Pearson's *r*), indicating how they were calculated

Our web collection on statistics for biologists contains articles on many of the points above.

Software and code

Policy information about availability of computer code

Data collection	Confocal fluorescence images were acquired using Olympus Zen Black Edition (ZEN 2.3 SP1 FP3 14.0.0.0). Light sheet images were acquired using Olympus Zen Black Edition (ZEN 2014 SP1 9.2.0.0). Wide-field fluorescence and bright-field images were acquired using Molecular Devices MetaMorph (versions 7.8.0.0 and 7.10.1.161). Finite-element modeling of protein mass transport was conducted using COMSOL Multiphysics (COMSOL 5.4).
Data analysis	3D renderings were generated using Olympus Zen Blue Edition (ZEN 2.3, ZEN lite 3.0). Images were brightness and contrast-adjusted, false-coloured, inverted, and cropped in Fiji (ImageJ 1.52n). All other data analysis (fluorescence quantification, separation peak fitting, migration distance and x-y and z-peak width quantification, contour plot generation, revolved intensity profile generation, separation lane segmentation, lysis monitoring, diffusional deconvolution, simulations in Fig. 3, cell settling quantification, experimental lysis monitoring quantification in Fig. 4 and comparison to simulated data, comparison of live-cell and probed fluorescence images) was conducted using custom scripts written in MATLAB (v2016b, v2018b).<br><br>All custom analysis code generated as a part of this work is available from the corresponding author upon reasonable request. Custom MATLAB scripts central to this work are deposited in a GitHub repository: https://github.com/samanthagrist/projection_ep_analysis.

For manuscripts utilizing custom algorithms or software that are central to the research but not yet described in published literature, software must be made available to editors/reviewers. We strongly encourage code deposition in a community repository (e.g. GitHub). See the Nature Research guidelines for submitting code & software for further information.

Data

Policy information about availability of data

All manuscripts must include a data availability statement. This statement should provide the following information, where applicable:

- Accession codes, unique identifiers, or web links for publicly available datasets
- A list of figures that have associated raw data
- A description of any restrictions on data availability

The datasets generated and analysed as a part of this work are available in the Dryad repository in the main data publication (DOI: 10.6078/D1B13V) and linked data publications (DOIs: 10.6078/D1N989, 10.6078/D1811G, 10.6078/D1HH6C). Source data for Figures 1(g), 2(c-e), 3(d-h), 4(e-g), 5(d-e, j-o) and Supplementary Figures 1, 4, and 6 are also provided as Supporting Data with the paper.

Field-specific reporting

Please select the one below that is the best fit for your research. If you are not sure, read the appropriate sections before making your selection.

☒ Life sciences ☐ Behavioural & social sciences ☐ Ecological, evolutionary & environmental sciences

For a reference copy of the document with all sections, see nature.com/documents/nr-reporting-summary-flat.pdf

Life sciences study design

All studies must disclose on these points even when the disclosure is negative.

Sample size

The research presents design, development, and 'proof of concept' performance of a new immunoassay format. Duplicate, triplicate, or quadruplicate experiments (each with >3 quantified separation lanes) are performed to establish immunoassay performance, following immunoassay development guidance (Assay Guidance Manual, Markossian S, Sittampalam GS, Grossman A, et al., editors.Bethesda (MD): Eli Lilly & Company and the National Center for Advancing Translational Sciences; 2004-, Chapter "Immunoassay Methods" by Karen L. Cox, BS, Viswanath Devanarayan, PhD, Aidas Kriauciunas, Joseph Manetta, BS, Chahrzad Montrose, PhD, and Sitta Sittampalam, PhD.)

Data exclusions

Separation lanes were excluded from the analysis if (1) the segmentation algorithm did not detect the fluorescent spot, or (2) the detected fluorescent spots did not meet the quality control criteria (signal-to-noise ratio >3, R-squared of Gaussian fit >0.7). The analysis scripts automatically included or excluded separation lanes based upon these criteria for both the purified protein and single-cell protein separation experiments (Fig. 1(g), Fig. 2(c-e), Fig. 3(d), Fig. 5(e, l-o), Supplementary Figures 1, 4, 5, 6). Additionally, lysis monitoring data were also excluded if the segmentation algorithm did not detect the fluorescent spot for the cell (Figure 4(e-f)).

Replication

All data reported in the main text were obtained from duplicate, triplicate, or quadruplicate gels. All attempts at replication of the data reported in the main text were successful. In the supplementary information (Supplementary Figure 6) we report beta tubulin migration distances in the same separation gel as actinin and GAPDH, imaged with both confocal and light sheet microscopy; however, for this dataset we report N=1 gel. The second gel showed high fluorescence background in confocal microscopy (leading to a low number of quantifiable separation lanes) and the second gel was not imaged in the 647 nm channel (for beta tubulin) with light sheet microscopy due to time constraints. Both gels were probed with Mouse anti-beta tubulin primary (GeneTex GTX11312, 1:15 dilution) and Donkey anti-Mouse AlexaFluor 647 secondary (Invitrogen A31571, lot 2045337, 1:10 dilution) antibodies.

As is typical in western blotting, performance is antibody-dependent. We screened the following antibody pairs and determined that each did not generate sufficient signal over the background to meet our data analysis criteria:
1. Rabbit anti-SFPQ (Abcam ab38148, 1:10 dilution)) primary and Donkey anti-Rabbit AlexaFluor 647 (Invitrogen A31573 lot 1964354, 1:20 dilution)
2. Mouse anti-lamin A/C mab636 (Invitrogen MA3-1000, 1:10 dilution) primary and Donkey anti-Mouse AlexaFluor 555 (Invitrogen A31570 lot 2045336, 1:20 dilution)
3. Mouse anti-HER2 (Abcam ab16901, lot GR3215414-16, 1:10 dilution) primary and Donkey anti-Mouse AlexaFluor 647 (Invitrogen A31571 lot 2045337, 1:10 dilution)

Randomization

As the goal of this work was to create and characterize a new measurement system, we did not have separate cell groups exposed to different conditions or protocols (only replicate gels).

Blinding

Blinding was not relevant to this study as the work did not include separate biological groups.

Reporting for specific materials, systems and methods

We require information from authors about some types of materials, experimental systems and methods used in many studies. Here, indicate whether each material, system or method listed is relevant to your study. If you are not sure if a list item applies to your research, read the appropriate section before selecting a response.

Materials & experimental systems

n/a	Involved in the study
☐	☒ Antibodies
☐	☒ Eukaryotic cell lines
☒	☐ Palaeontology
☒	☐ Animals and other organisms
☒	☐ Human research participants
☒	☐ Clinical data

Methods

n/a	Involved in the study
☒	☐ ChIP-seq
☒	☐ Flow cytometry
☒	☐ MRI-based neuroimaging

Antibodies

Antibodies used	Primary antibodies used were Rabbit α-actinin (Cell Signaling Technologies 6487, lot 2), Mouse α-PTBP1 (Sigma WH0005725M1, lot J4241-3H8), and Goat α-GAPDH (Sigma SAB2500450, lot 6377C3); secondary antibodies were Donkey α-Rabbit AF555 (Invitrogen A-31572, lot 2017396), Donkey α-Mouse AF555 (Invitrogen A-31570, lot 2045336), and Donkey α-Goat AF488 (Invitrogen A-11055, lot 2059218). Bio-Rad hFAB probes for GAPDH (12004167, control 64225941) and tubulin (12004165, control 64210670) were used for initial system characterization. Antibody dilutions are reported in Table 2 of the manuscript.
Validation	Cell Signaling Technologies 6487 was validated by the manufacturer. Validation steps include "Examination of several cell lines and/or tissues of known expression levels allows accurate determination of species cross-reactivity and verifies specificity. Treatment of cell lines with growth factors, chemical activators or inhibitors, which induce or inhibit target expression, verifies specificity. Phosphatase treatment confirms phospho-specificity. The use of siRNA transfection or knockout cell lines verifies target specificity. Side-by-side comparison of lots to ensures lot-to-lot consistency. Optimal dilutions and buffers are predetermined, positive and negative cell extracts are specified, and detailed protocols are already optimized, saving valuable time and reagents." Source: https://www.cellsignal.com/contents/antibody-validation-principles/antibody-validation-for-western-blotting/ourapproach-validation-western-blot#:~:text=Cell%20Signaling%20Technology%20(%20CST%20)%20provides,according%20to%20a%20rigorous%20protocol. Additionally, our group has used this antibody in previous published studies involving protein separation (dois: 10.1038/s41698-018-0052-3, 10.1039/C9LC00917E).<br><br>Sigma WH0005725M1 was validated by the manufacturer (for Western blotting and ELISA). Additionally, this antibody has been previously used and published for protein expression quantification via immunostaining (doi: 10.1101/gr.178426) and for western blotting (doi: 10.1038/nprot.2013.020) and immunofluorescence (doi: 10.1016/j.molcel.2019.01.017).<br><br>Sigma SAB2500450 was validated by the manufacturer (for Western blotting and ELISA). Additionally, our group has used this antibody in several previous studies involving protein separation and observed no specificity issues (dois: 10.1038/s41698-018-0052-3, 10.1039/C9LC00917E, 10.1002/smll.201802865, 10.1039/c6lc01333c, 10.1021/acs.analchem.7b03096, 10.1038/ncomms14622).<br><br>GAPDH and tubulin hFAB probes (Bio-Rad 12004167, 12004165) were validated by the manufacturer (testing for target protein detection, sensitivity, and cross-reactivity specifications). Additionally, their use has been previously reported in the peer-reviewed literature for western blotting (dois: 10.1016/j.celrep.2019.05.004, 10.1038/s41467-019-13314-y, 10.7554/eLife.38319, 10.1371/journal.pone.0209833, 10.1002/cpph.72)<br><br>All experiments performed size-based protein separations, in which off-target bands of larger or smaller molecular weight would be visible.

Eukaryotic cell lines

Policy information about cell lines

Cell line source(s)	BT474 breast tumour cells were purchased from the UC Berkeley Biosciences Divisional Services Cell Culture Facility. U251 human glioblastoma cells stably transduced with turboGFP by lentiviral infection were kindly provided by Prof. Sanjay Kumar's laboratory at UC Berkeley (the naïve, pre-modification U251 cell line was obtained from the UC Berkeley Tissue Culture Facility, originally sourced from the American Type Culture Collection)."
Authentication	BT474s authenticated by Short Tandem Repeat with 100% match to BT474 in October 2019. U251-turboGFP cells were authenticated by Short Tandem Repeat with 100% match to U251 in October 2019. Authentication was performed by the UC Berkeley Cell Culture Facility.
Mycoplasma contamination	All cell lines tested negative for mycoplasma contamination October 31, 2019 (as well as in 2018). Testing was performed by the UC Berkeley Cell Culture Facility. Cells were fixed with methanol and stained with Hoechst nuclear stain to visualize mycoplasma nuclei within the cell membrane.
Commonly misidentified lines (See ICLAC register)	Commonly misidentified cell lines were not used in this study.
